# Tyrosine-mutated AAV2-mediated shRNA silencing of PTEN promotes axon regeneration of adult optic nerve

**DOI:** 10.1371/journal.pone.0174096

**Published:** 2017-03-21

**Authors:** ZhengRu Huang, ZiZhong Hu, Ping Xie, QingHuai Liu

**Affiliations:** 1 Department of Ophthalmology, the First Affiliated Hospital of Nanjing Medical University, Nanjing, Jiangsu Province, China; 2 Department of Ophthalmology, the Second People´s Hospital of Changshu, Changshu, Jiangsu Province, China; Dalhousie University, CANADA

## Abstract

Activating PI3K/AKT/mTOR signaling pathway via deleting phosphatase and tensin homolog (PTEN) has been confirmed to enhance intrinsic growth capacity of neurons to facilitate the axons regeneration of central nervous system after injury. Considering conditional gene deletion is currently not available in clinical practice, we exploited capsid residue tyrosine 444 to phenylalanine mutated single-stranded adeno-associated virus serotype 2 (AAV2) as a vector delivering short hairpin RNA to silence PTEN to promote retinal ganglion cells (RGCs) survival and axons regeneration in adult rat optic nerve axotomy paradigm. We found that mutant AAV2 displayed higher infection efficiency to RGCs and Müller cells by intravitreal injection, mediated PTEN suppression, resulted in much more RGCs survival and more robust axons regeneration compared with wild type AAV2, due to the different extent of the mTOR complex-1 activation and glutamate aspartate transporter (GLAST) regulation. These results suggest that high efficiency AAV2-mediated PTEN knockdown represents a practicable therapeutic strategy for optic neuropathy.

## Introduction

Like other mammalian mature central nerve system (CNS) neurons, retinal ganglion cells (RGCs) are normally unable to regenerate axons spontaneously after optic nerve injury, which causes irreversible vision loss. The failure of axons regeneration has been attributed to the apoptosis of RGCs, insufficient intrinsic growth capacity of mature neurons, lack of suitable stimuli, and inhibitory extracellular environment [1–2]. In the past decade, many evidences have supported that activating the intrinsic growth capacity is able to induce a robust regenerative response in mature axotomized RGCs [3–4]. Deletion of phosphatase and tensin homolog (PTEN), which is a negative regulator of mammalian target of rapamycin (mTOR), have been demonstrated to wake up the regenerative ability of adult corticospinal neurons and RGCs [5–6]. However, conditional gene deletion is currently impossible to translate to clinical practice, while therapies based on small-interfering RNA (siRNA) to knockdown target genes may be the most useful strategy for the treatment of optic neuropathy [7].

The eye has been considered a suitable target for gene therapy mainly because (1) it is a relatively small closure compartment, permitting local delivery of siRNAs by direct injection [7–8]. (2) It is under a relatively immune-privileged status due to the absence of antigen-presenting cells and the presence of factors that actively suppress T cell activation, which facilitates stable expression of the target transgene [9]. Thus, gene therapy has been well established to treat ocular diseases such as retinitis pigmentosa and Leber's congenital disease [10–11]. Adeno-associated virus (AAV) of different serotype has been considered suitable vector for gene delivery, achieving safe, efficient, and long-term transgene expression [12]. Despite AAV serotype 2 (AAV2) has been shown to effectively transduce post-mitotic neurons, including the RGCs preferentially via intravitreal injection [13–14], it takes too long (6–8 weeks) post-injection to reach maximal gene expression in the retina to rescue RGCs in traumatic optic neuropathy, a disease characterized by quick deterioration of RGCs [15]. Therefore, improved AAV vectors are needed to expedite the transgene expression with high level. It has been reported that site-directed tyrosine (Y) to phenylalanine (F) mutation of capsid surface-exposed and highly conserved tyrosine residues is able to dramatically increase the transduction efficiency of self-complementary AAV2 (scAAV2) following intraocular injection [16–17].

The purpose of the present study was to evaluate the intraocular transduction of the single point Y 444 to F mutated single-stranded AAV2 (Y444F ssAAV2) via intravitreal injection, and exploited it as a vector delivering short hairpin RNA silencing PTEN to promote RGCs survival and axons regeneration after optic nerve axotomy (ONA). Our data revealed a significantly increased transduction efficiency of Y444F ssAAV2 to RGCs and especially to Müller cells. Compared with wild type (Wt) AAV2, Y444F AAV2-mediated PTEN knockdown could lead to more RGCs survival and more robust axons regeneration back to optic chiasm after axotomy in wild-type rats, which presented a translatable treatment strategy for traumatic optic neuropathy.

## Materials and methods

### Animals

Adult female Sprague-Dawley rats (200–250 g), purchased from SLRC (Shanghai, China), were used for all experiments, and raised in Makrolon cages with a 12 h:12 h light-dark cycle. Food and water were provided ad libitum. This study was carried out in strict accordance with the recommendations in the Guide for the Care and Use of Laboratory Animals of the National Institutes of Health. The protocol was approved by the Committee on the Ethics of Animal Experiments of Nanjing Medical University. All surgeries were performed under deep anesthesia with intraperitoneal injection of 3% sodium pentobarbital (50 mg/kg body weight). Rats were kept warm up to waking up from anesthesia, sacrificed by giving a lethal overdose of anesthesia. All efforts were made to minimize suffering.

### Production of AAV2

**Fig 1:**
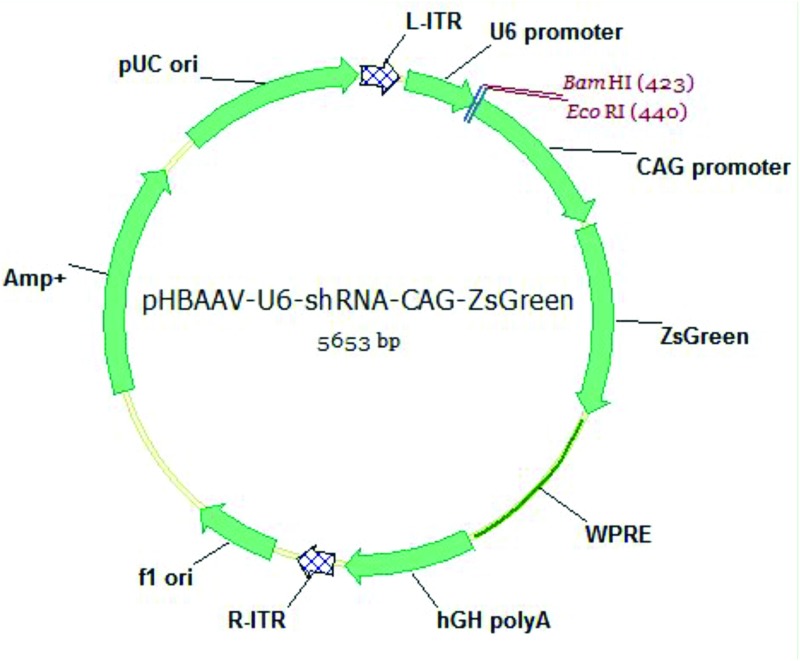
A map of AAV2 construct used to knockdown PTEN. The shRNA.PTEN was inserted into the gap between BamHI and EcoRI, expressed under the control of an U6 promoter. A robust and non-cell-specific chicken β-actin (CBA) promoter controlled the expression of ZsGreen acting as control reporter.

The most effective shRNA targeting PTEN was selected based on previous report [18]. The sequence of this shRNA was cloned in the vector pAAV-U6-CAG-ZsGreen as described before [19] (Fig 1). In brief, 1μl of the forward primer and 1μl of the reverse primer containing the shRNA sequence were dissolved in 16μl water and 2μl of 10×annealing buffer (100 mM Tris, pH 7.5, 1M NaCl and 10 mM EDTA). The solution was boiled for 5 min and then cooled to room temperature. Annealed oligos were ligated to BamH I/EcoR I-cut backbone fragment of pAAV-U6-CAG-ZsGreen. The sequence of the shRNA primers was as follows (in bold: sequence of siRNA-sense and siRNA-antisense strand, in italics: sequence of the hairpin turns). The forward primer: 5’-GATCC**GGCACTGTTGTTTCACAAGAT***TTCAAGAGA***ATCTTGTGAAACAACAGTGCC**TTTTTTC-3’; The reverse primer: 5’-AATTGAAAAAA**GGCACTGTTGTTTCACAAGAT***TCTCTTGAA***ATCTTGTGAAACAACAGTGCC**G-3’. The plasmid was sequenced to confirm its correct identity.

Production of wild-type (Wt) AAV2-GFP or Wt AAV2-shRNA. PTEN-GFP were performed as reported before [20]. Briefly, HEK293 cells were transfected with calcium phosphate, HEPES-buffered saline and a serotype specific plasmid complex containing 10μg pAAV-RC (Stratagene, La Jolla, CA, USA), 20μg pHelper (Stratagene, La Jolla, CA, USA) and 10μg pAAV-GFP or pAAV-shRNA.PTEN-GFP plasmid. Single site-directed mutagenesis of surface-exposed tyrosine residue on AAV2 VP3 was performed as previously described [17]. Y444F ssAAV2-shRNA.PTEN-GFP was produced by co-transfection with mutated-pAAV-RC plasmid encoding capsid containing point mutation of surface exposed tyrosine residue 444 to phenylalanine. Seventy-two hours after transfection, cells were harvested and AAV were purified by dialysis and virus gradient centrifugation in iodixanol. Protein liquid chromatography was performed to obtain high titer viral stocks. The viral titers were determined using qPCR and normalized to 1.0 × 10^12^ viral genomes per milliliter (vg/ml) using balanced salt solution.

### Intravitreal injection

AAV vectors (5μl, titers at 1.0 x 10^12^) or cholera toxin β subunit conjugated fluorescein isothiocyanate (CTB-FITC) (5μl of 0.2%, Sigma, Saint Louis, MO, USA) were injected intravitreally using 5-μl Hamilton syringe (Hamilton, Bonaduz, Switzerland) [21]. Briefly, the needle was inserted in peripheral retina, just behind the ora serrata, and was carefully angled and located to avoid unexpected damage. Rats with cataract, retinal detachment, or vitreous hemorrhage were excluded from this study.

### Retrograde labeling of RGCs by stereotactical injections

To visualize RGCs, FluoroGold (Sigma, Saint Louis, MO, USA) was injected stereotactically into the superior colliculus 5 days before sacrifice as previous report [22]. The skull was exposed with a midline incision and two small holes were drilled 6.5 mm posterior to bregma and 1.8 mm lateral to the midline on both sides. The needle was inserted 3.6 mm into the brain, and then 2 μl of 5% FluoroGold was injected stereotactically using 5-μl Hamilton syringe. The skin incision was sutured with 5–0 silk thread.

### Optic nerve axotomy

ONA of right eyes was performed reportedly [22–23]. In brief, the lateral canthus was incised along the orbital rim and the lacrimal gland was moved to the side. The eyeball was slightly rotated by pulling the superior rectus muscle. The optic nerve was then exposed intraorbitally, and crushed with jeweler’s forceps (Dumont #5; Roboz) at a distance of at least 2 mm behind eyeball for 30 seconds approximately, avoiding damage to the ophthalmic artery. The vascular integrity of the retina was examined by fundoscopy. Rats in which the retinal vessel was injured or questioned were excluded from this study.

### Examination of immunofluorescence

Rats were given a lethal overdose of anesthesia and perfused transcardially with 4% paraformaldehyde (PFA). Eyes were post-fixed in the same fixative, cryoprotected in 30% sucrose solution overnight at 4°C, and frozen in optimal cutting temperature compound (Tissue Tek). For immuno-staining of phospho-S6 ribosomal protein (pS6), PTEN, and glutamine synthetase (GS), longitudinal frozen sections of eyes were cut at 8 μm thickness. For quantifying the density of RGCs, whole retinas were dissected out. Frozen sections were blocked with immuno-staining blocking buffer (Beyotime, Shanghai, China) and permeabilized with 0.2% Triton X-100 for 1 hour at room temperature. Then the sections were incubated overnight at 4°C with anti-pS6 antibody (1:100, Cell Signaling Technology, Danvers, MA, USA), anti-PTEN antibody (1:125, Cell Signaling Technology, Danvers, MA, USA), anti-GS antibody (1:250, Abcam, Cambridge, MA, USA). Retinas were blocked with immuno-staining blocking buffer and permeabilized with 0.2% Triton X-100 for 2 hours at room temperature. Then the retinas were immunostained overnight at 4°C with neuronal class III β-tubulin (TUJ1) antibody (1:250, Beyotime, Shanghai, China), which specifically labels adult RGCs. The sections or retinas were rinsed with 0.1M phosphate buffer saline for 5 min for three times and then incubated with secondary antibody conjugated cy3 (1:500, Beyotime, Shanghai, China) for 1 hour at room temperature. Washed for 5 min for three times, the sections or retinas were examined under the fluorescent microscope (Nikon Eclipse50i, Japan) to capture images with a CCD camera with the same parameters respectively.

### Quantitation of RGCs

The retinas immuno-stained with TUJ1 antibody, or retrogradely labeled with FluoroGold were mounted onto pre-coated glass slides, and the images were captured under the fluorescent microscope with a CCD camera. Sixteen fields in middle area of retinas (about 0.276mm^2^ per field at 100×magnification), radially distributed at 1mm to 2 mm from the optic nerve disc, were sampled per retina. The total TUJ1 positive cells or the total FluoroGold-labeled and GFP positive cells at the same field in each image were counted, and then the density of TUJ1 positive RGCs or the percentage of GFP positive cells in FluoroGold-labeled cells was obtained.

### Evaluation of immunofluorescence of PTEN

The average PTEN fluorescent intensity in retinal ganglion layer (GCL) or inner nuclear layer (INL) was measured and averaged based on 3 images per section, 2 non-consecutive sections per eye using ImageJ software (National Institute of Health, Bethesda, MD, USA). The entire GCL or INL of each image was selected for measurement. Individual mean values were then averaged across each group.

### Measurement of pS6 immunoreactivity in GCL

Immuno-labeled sections were counter-stained with 4', 6-diamidino-2-phenylindole (DAPI) before images capturing. pS6 positive cells and DAPI-stained cells were counted in entire GCL in each image. The percentage of pS6 positive cells in total DAPI-stained cells was obtained. Values for pS6 were based on 3 images per section, 2 non-consecutive sections per eye, and then averaged for mean values across each group.

### Western blotting assay

Total protein of retina was collected and the concentration was quantified using BCA protein assay kit (Beyotime, Shanghai, China). Protein samples (30μg) were loaded and separated on 10% SDS-polyacrylamide gel and then transferred to polyvinylidene fluoride membranes (0.22μm pore; Millipore, Billerica, MA, USA). The primary antibodies used were anti-PTEN antibody (Cell Signaling Technology, 1:1000, Danvers, MA, USA), anti- glutamate-aspartate transporter (GLAST) antibody (Abcam, 1:2500, Cambridge, MA, USA), anti-pS6 antibody (Cell Signaling Technology, 1:2000, Danvers, MA, USA), anti-glyceraldehyde-3-phosphate dehydrogenase (GAPDH) antibody (Beyotime, 1:5000, Shanghai, China). Secondary antibody (Beyotime, 1:1000, Shanghai, China) was horseradish peroxidase-conjugated. The immune-complexes were detected by enhanced chemiluminescence (Millipore, Billerica, MA, USA). Blots were quantified by densitometry and normalized by use of GAPDH to correct the differences in loading of the proteins. For densitometric analyses, the bands were quantified using Image Lab software (BioRad laboratories, Hercules, CA, USA). Each experiment was performed at least three times.

### Assessing for regenerating axons

To visualize and quantify regenerating axons of RGCs, 5 μl of 0.2% CTB-FITC was injected into the vitreous for anterograde labeling the visual pathway 3 days before sacrifice. The orbital optic nerve segments, the optic chiasms, and the brains were dissected out, post-fixed in 4% PFA, and transferred to 30% sucrose solution overnight at 4°C. Longitudinal frozen sections of optic nerves, coronal frozen sections of optic chiasms and brains were cut at 8μm, 10μm, and 14μm thickness respectively, and mounted onto pre-coated glass slides. At least 5 non-consecutive sections were captured under the fluorescent microscope for each animal with the same parameters respectively. Fluorescent intensity of CTB-FITC of optic nerve at different distances from the ONA site were analyzed with ImageJ software.

### Statistics

All data were displayed as mean ± standard difference (SD). One-way analysis of variance followed by Bonferroni test was used to compare multiple groups. Pairwise comparison between groups was performed using the Student’s *t*-test. *P*<0.05 or lower was considered statistically significant. Statistical analysis was carried out using Stata11.4 software.

## Results

### Confirmation of complete axotomy

**Fig 2:**
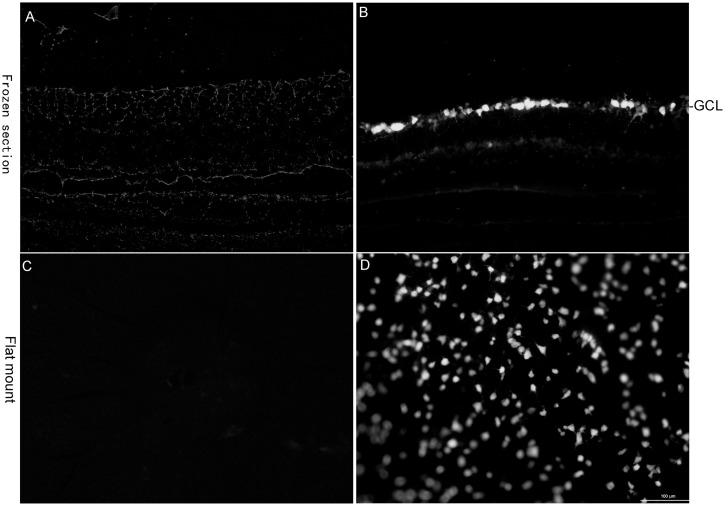
FluoroGold retrograde labeling of RGCs identifying the complete axotomy. No RGCs was labeled with FluoroGold in retinal longitudinal sections (A) and whole-mounts (C) of axotomized rats while numerous RGCs were labeled with FluoroGold in retinal longitudinal sections (B)and whole-mounts (D) of intact rats. Scale bar, 100μm.

Normally, the FluoroGold fluorescence was strong in frozen sections and flat mounts from intact control group. After ONA, however, no FluoroGold positive cells were found in frozen sections and flat mounts (n = 5) (Fig 2). The success of ONA was verified by the failure of retrograde tracer to reach the retina after crush.

### Efficiency of transgene expression of AAV2 vectors

**Fig 3:**
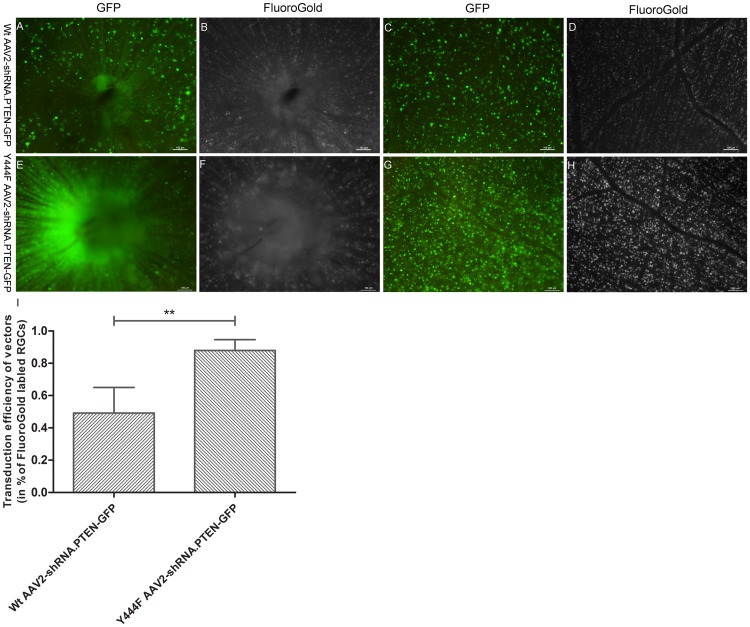
FluoroGold retrograde labeling evaluating the transduction efficiency of AAV2 vectors 4 weeks after injection. Retinal whole-mounts displayed FluoroGold-labeled (white) RGCs and gene-transduced (green) RGCs from the same retinal regions of rats intravitreally injected Wt AAV2-shRNA.PTEN-GFP or Y444F AAV2-shRNA.PTEN-GFP vector (A-H). Quantifying the percentage of GFP-positive cells in FluoroGold-labeled cells revealed the transduction capacity of Y444F AAV2-shRNA.PTEN-GFP was significantly stronger than that of Wt AAV2-shRNA.PTEN-GFP (I). ** *P* < 0.01 as tested by Student’s *t*-test. Scale bar, 100μm.

To evaluate the efficiency of transgene expression, Wt AAV2-shRNA.PTEN-GFP and Y444F AAV2-shRNA.PTEN-GFP vector were injected into intact eyes of rats containing normal RGCs population. The density of RGCs expressing GFP was measured in retinal flat mounts after 4 weeks, and was compared to the density of RGCs labeled retrogradely with FluoroGold in the same area. Wt AAV2 showed a widespread GFP fluorescence, with moderate transgene efficiency (n = 6) (Fig 3A and 3C), while Y444F AAV2 showed a widespread GFP fluorescence and much higher transgene efficiency compared with Wt AAV2 (n = 6) (Fig 3E and 3G). Quantitative assessment indicated more cells were transduced with mutant vector than with Wt vector (87.93 ± 6.68% *vs* 49.19 ± 15.81%) (n = 6) (Fig 3I). These results demonstrated that Y444F mutation could significantly enhance the transgene efficiency of ssAAV2.

### Target cells of transgene expression of AAV2 vectors

**Fig 4:**
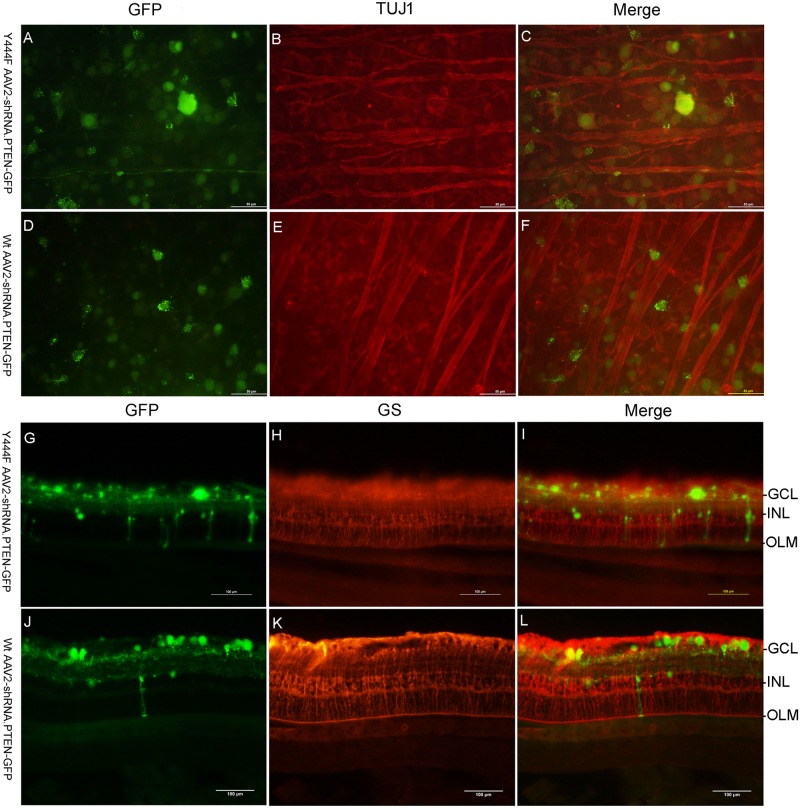
Immunofluorescence displaying RGCs and Müller cells transgene expressing GFP 4 weeks after intravitreal injection. Merged image showed colocalization of GFP fluorescence and TUJ1 staining in retinal flat-mounts from eyes treated with Y444F AAV2-shRNA.PTEN-GFP (A-C) or Wt AAV2-shRNA.PTEN-GFP (D-F), showed colocalization of GFP fluorescence and GS staining in retinal sections from eyes treated with Y444F AAV2-shRNA.PTEN-GFP (G-I) or Wt AAV2-shRNA.PTEN-GFP (J-L). Scale bar, 50μm.

To verify the target cells of transduction of Wt or mutant AAV2 after intravitreal injection, histological analysis was performed with retinal flat mounts and frozen sections. In flat mounts, cells expressed GFP were stained with TUJ1 antibody (n = 5) (Fig 4A–4F). In frozen sections of eyes treated with Wt AAV2-shRNA.PTEN-GFP, GFP-expressing cells were detected mainly in GCL and occasionally in INL (n = 5) (Fig 4J). In eyes injected Y444F AAV2-shRNA.PTEN-GFP, GFP-expressing cells were detected in both GCL and INL, although the number of GFP-expressing cells in INL was less than that in GCL, but appeared to be much greater than that in eyes injected Wt AAV2-shRNA.PTEN-GFP (n = 5) (Fig 4G). The majority of cells expressing GFP in INL spanned nearly the full thickness of the retina, from the GCL to the outer limiting membrane (OLM), which matched the histological feature of Müller cells [24]. Further immunofluorescent identification confirmed that these cells were also immune-stained with GS antibody (n = 5) (Fig 4I and 4L). These results revealed that both Wt AAV2 and Y444F AAV2 could transduce RGCs. Only could Y444F AAV2 efficiently transduce RGCs and Müller cells simultaneously.

### Expression of PTEN, pS6, GLAST

**Fig 5:**
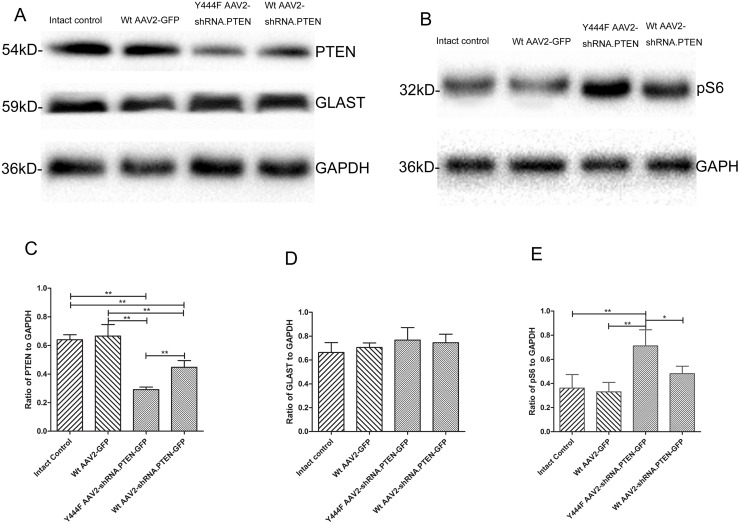
Western-blot assessing the expression of PTEN, pS6, and GLAST in retina 4 weeks after intravitreal injection of AAV2 vectors. Expression of PTEN in retinas transduced with Y444F AAV2-shRNA.PTEN or Wt AAV2-shRNA.PTEN significantly decreased compared with Wt AAV2- GFP or intact control, and that the knockdown extent with Y444F AAV2-shRNA.PTEN was higher than that with Wt AAV2-shRNA.PTEN (A, B). As a result, transduction with Y444F AAV2-shRNA.PTEN significantly promoted the expression of pS6 (A, C). Levels of GLAST were not altered in the retinas transduced with any kinds of vectors compared with intact control (A, D). *P < 0.05, **P < 0.01 in ANOVA followed by Bonferroni’s post-test.

**Fig 6:**
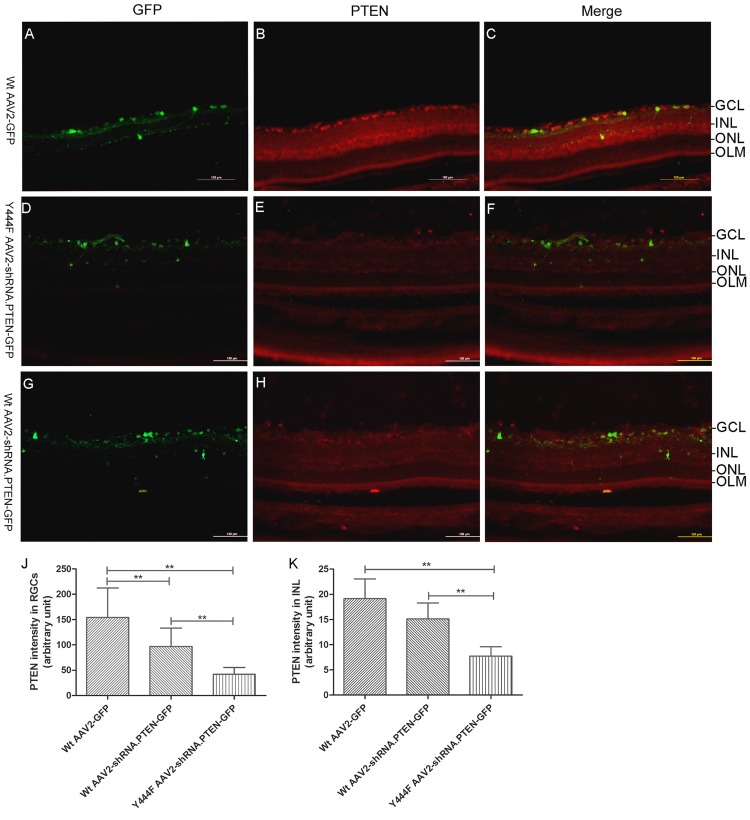
Immunofluorescence testing PTEN in retinas 4 weeks after intravitreal injection of AAV2 vectors. Immunofluorescence of retinal sections showed PTEN expression in retinas following Wt AAV2-GFP (A-C), Y444FA AV2-shRNA.PTEN (D-F), or Wt AAV2-shRNA.PTEN (G-I) injection. Quantification of PTEN expression, measured by ImageJ densitometry method, revealed a significant difference in GCL and INL treated with Wt AAV2-GFP, Wt AAV2-shRNA.PTEN, and Y444F AAV2-shRNA.PTEN respectively (J). **P < 0.01 in ANOVA followed by Bonferroni’s post-test. Scale bar, 100μm.

Western blotting assay and/or immunohistochemistry were used to test the expression of PTEN, pS6, and GLAST 4 weeks after intravitreal injection. Western blotting showed that compared with Wt AAV2-GFP (0.66 ± 0.08) or intact control (0.64 ± 0.04), there was a significant decrease of PTEN expression in both Y444F AAV2-shRNA.PTEN (0.29 ± 0.02) or Wt AAV2-shRNA.PTEN (0.45 ± 0.05) (n = 5) (Fig 5A and 5C), and that the reduction was much more significant in Y444F AAV2-shRNA.PTEN-treated rats than in Wt AAV2-shRNA.PTEN-treated rats (55% *vs* 29%). Immunofluorescence of PTEN in GCL showed a significant difference in Wt AAV2-GFP (154.92 ± 57.41), Wt AAV2-shRNA.PTEN (97.00 ± 36.22), and Y444F AAV2-shRNA.PTEN (42.07 ± 13.49) groups (n = 5) (Fig 6J). The fluorescence intensity of PTEN in INL treated with Y444F AAV2-shRNA.PTEN was also significantly lower than that treated with Wt AAV2-shRNA.PTEN or Wt AAV2-GFP (n = 5) (Fig 6k). These results indicated that Y444F AAV2-shRNA.PTEN suppressed the expression of PTEN not only in GCL but also in INL, which was in accord with the transduction of RGCs and Müller cells.

**Fig 7:**
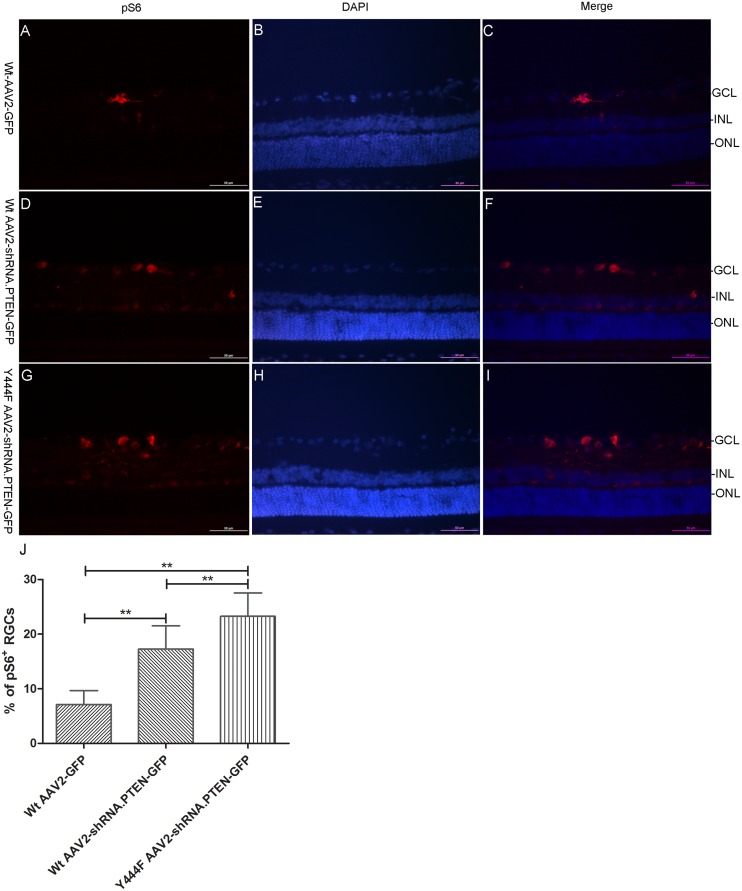
pS6 immunoreactivity in retinas 4 weeks after AAV2 vectors intravitreal injection. Immunofluorescence of sections showed pS6 positive cells in retina following Wt AAV2-GFP (A-C), Wt AAV2-shRNA.PTEN (D-F), or Y444F AAV2-shRNA.PTEN (G-I) injection. Quantifying the percentage of pS6 positive cells in GCL indicated a significant difference in Wt AAV2-GFP, Wt AAV2-shRNA.PTEN, and Y444F AAV2-shRNA.PTEN-treated retina (J). The fluorescence of pS6 also presented in inner nuclear layer (INL) of Y444F AAV2-shRNA.PTEN-treated retinas, which was almost absent in Wt AAV2-shRNA.PTEN or Wt AAV2-GFP-treated retinas. **P < 0.01 in ANOVA followed by Bonferroni’s post-test. Scale bar, 50μm.

The most widely used biochemical marker of mTOR complex-1 activity is pS6 [5], thus the expression of pS6 was evaluated 4 weeks after injection. As expected, Western blotting founded that the expression of pS6 in retinas transduced with either Wt AAV2-shRNA.PTEN (0.48 ± 0.06) or Y444F AAV2-shRNA.PTEN (0.71 ± 0.13) was significantly increased compared with Wt AAV2-GFP (0.33 ± 0.08) or intact control (0.36 ± 0.11). Quantitation showed that transduction with Wt AAV2-shRNA.PTEN or Y444F AAV2-shRNA.PTEN augmented approximate 45%, 115% respectively in pS6 expression compared to Wt AAV2-GFP (n = 5) (Fig 5B and 5E). The analysis for the percentage of pS6 positive cells in GCL also revealed the significant difference in Wt AAV2-GFP (7.08 ± 2.57%), Wt AAV2-shRNA.PTEN (17.25 ± 4.26%), and Y444F AAV2-shRNA.PTEN (23.25 ± 4.31%) groups. In addition, pS6 positive cells were also presented in INL of Y444F AAV2-shRNA.PTEN-treated retinas, which was almost absent in Wt AAV2-shRNA.PTEN or Wt AAV2-GFP-treated retinas. (n = 6) (Fig 7).

**Fig 8:**
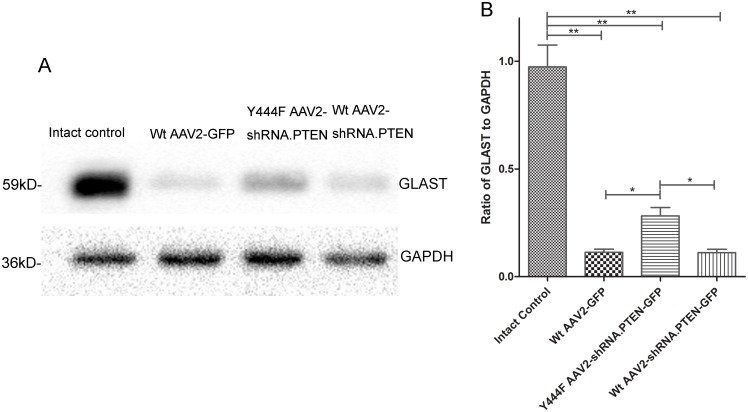
Western-blotting for the expression of GLAST in retina 6 weeks after axotomy. Compared to intact control, ONA resulted in dramatic down-regulation of GLAST, yet Y444F AAV2-shRNA.PTEN, compared with Wt AAV2-shRNA.PTEN or Wt AAV2-GFP, inhibited the reduction significantly. *P < 0.05, **P < 0.01 in ANOVA followed by Bonferroni’s post-test.

In consideration of that Y444F AAV2-shRNA.PTEN injected intravitreally could infect Müller cells, the GLAST expression was next analyzed. Although the GLAST expression of each group 4 weeks after injection had no statistical difference (n = 5) (Fig 5A and 5D), a dramatical decrease of GLAST expression in Y444F AAV2-shRNA.PTEN (0.28 ± 0.04), Wt AAV2-shRNA.PTEN (0.12 ± 0.02), and Wt AAV2-GFP group (0.11 ± 0.01) was shown compared with the intact control group (0.97 ± 0.10) 6 weeks after axotomy, but the expression of GLAST in Y444F AAV2-shRNA.PTEN group was still higher than that of Wt AAV2-shRNA.PTEN, or Wt AAV2-GFP group (n = 5) (Fig 8A and 8B). These findings indicated that axotomy decreased the expression of GLAST in retinas and Y444F AAV2-shRNA.PTEN reduced the decrease of GLAST expression caused by axotomy.

### Survival of RGCs

**Fig 9:**
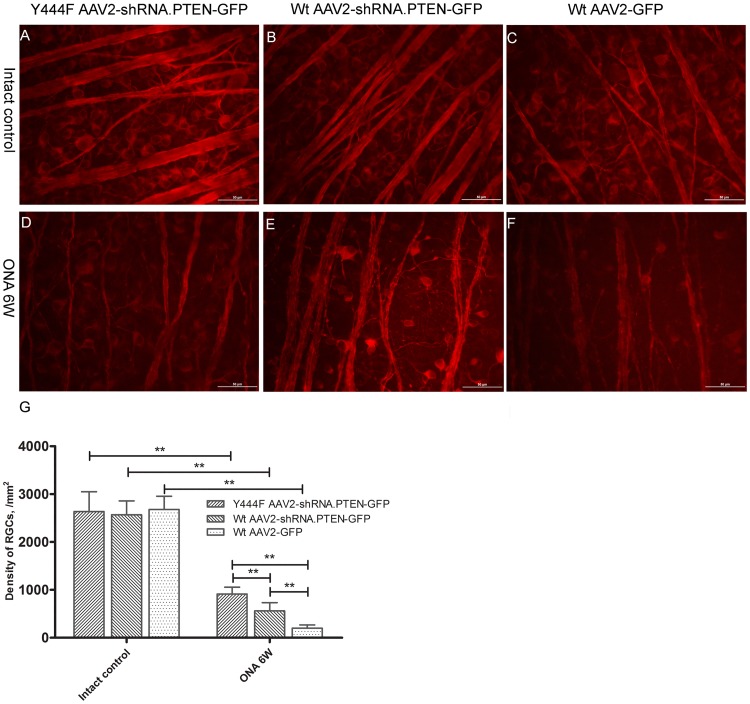
TUJ1 immuno-labeling evaluating RGCs survival 6 weeks after axotomy. The number of survived RGCs decreased significantly 6 weeks after axotomy. Compared with Wt AAV2-GFP, both Y444F AAV2-shRNA.PTEN and Wt AAV2-shRNA.PTEN significantly prompted RGCs survival, and the pro-survival effect of Y444F AAV2-shRNA.PTEN was stronger than that of Wt AAV2-shRNA.PTEN. **P < 0.01 in Student’s *t*-test or ANOVA followed by Bonferroni’s post-test. Scale bar, 50μm.

To evaluate pro-survival effect of AAV2-shRNA.PTEN on RGCs, AAV2 vectors was injected intravitreally 4 weeks before ONA. RGCs were identified by immune-labeling with TUJ1 antibody 6 weeks after ONA. The density of immune-stained RGCs in middle region of the retina was quantified on flat mounts (n = 5) (Fig 9). ONA resulted in considerably RGCs loss in each group 6 weeks after ONA. The number of survived RGCs in retinas transduced with Wt AAV2-shRNA.PTEN (561 ± 170) was significantly increased 6 weeks after axotomy compared with that in retinas transduced with Wt AAV2-GFP (201 ± 65) and the survival was further improved in retinas transduced with Y444F AAV2-shRNA.PTEN (912 ± 144). In addition, the collapse of retinal nerve fibers treated with either Wt AAV2-shRNA.PTEN or Y444F AAV2-shRNA.PTEN vector seemed less serious compared with that of treated with Wt AAV2-GFP vector.

### Regeneration of axons

**Fig 10:**
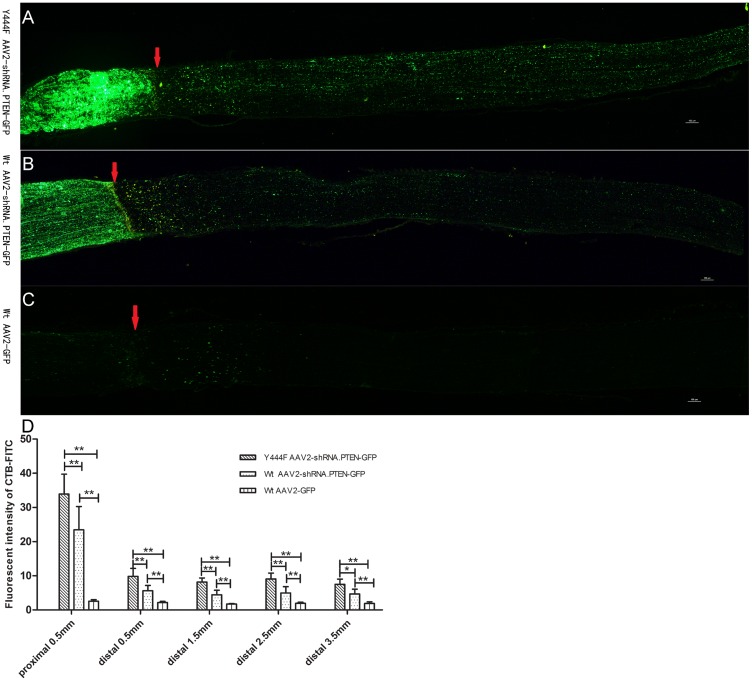
PTEN knockdown promoting axons regeneration in optic nerve 6 weeks after axotomy. Fluorescent images of optic nerve longitudinal sections showed CTB-FITC labeled regenerating axons of rats treated with Y444F AAV2-shRNA.PTEN (A), Wt AAV2-shRNA.PTEN (B), and Wt AAV2-GFP (C) respectively. Quantification of the fluorescence intensity at different distances proximal to and distal to the ONA site showed the significant difference in Y444F AAV2-shRNA.PTEN, Wt AAV2-shRNA.PTEN and Wt AAV2-GFP groups (D). *P < 0.05, **P < 0.01 in ANOVA followed by Bonferroni’s post-test. Scale bar, 100μm. Arrow, ONA site.

**Fig 11:**
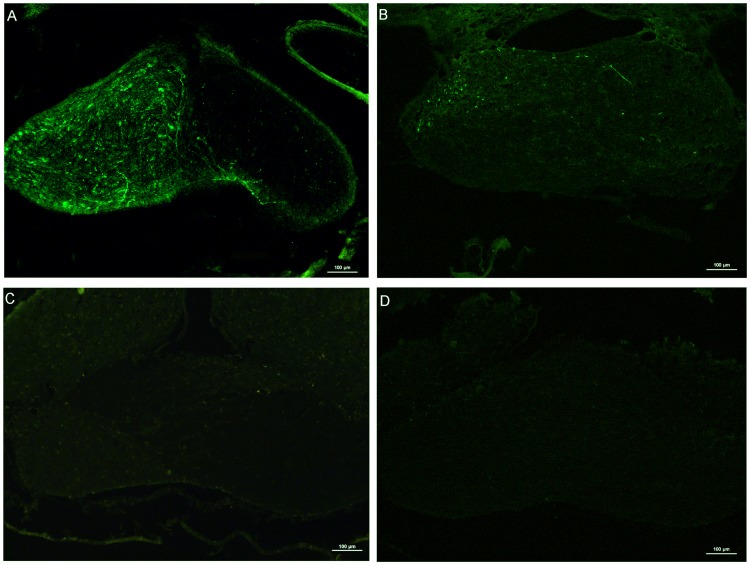
Regenerating axons in optic chiasm labeled with CTB-FITC 6 weeks after axotomy. Fluorescent images of optic chiasm coronal sections displayed normal CTB-FITC-labeled axons of intact rats (A), considerable regenerating axons of Y444F AAV2-shRNA.PTEN-treated rats (B), no regenerating axons of Wt AAV2-shRNA.PTEN-treated rats (C) or Wt AAV2-GFP-treated rats (D). Scale bar, 100μm.

In addition to evaluating the RGCs survival of AAV2 vectors after ONA, we also investigated the axons regeneration. In rats transduced with Wt AAV2-GFP, few regenerating neurites crossed the lesion site and no neurites could be observed over 500μm distal to the ONA site (n = 5) (Fig 10c). In contrast, transduction with Y444F AAV2-shRNA.PTEN or Wt AAV2-shRNA.PTEN led to axons regeneration extending over 4 mm from the lesion site (n = 5) (Fig 10A and 10B). Quantifying for fluorescent intensity of CTB-FITC showed a significant difference in Y444F AAV2-shRNA.PTEN, Wt AAV2-shRNA.PTEN and Wt AAV2-GFP groups (n = 5) (Fig 10D). Regenerating axons were not observed in the optic chiasm of Wt AAV2-shRNA.PTEN (n = 5) (Fig 11c) or Wt AAV2-GFP (n = 5) (Fig 11d) groups. On the contrary, considerable regenerating axons were found in optic chiasm of Y444F AAV2-shRNA.PTEN-treated rats (n = 5) (Fig 11B), although the number of regenerating axons were less than that of intact control (n = 5) (Fig 11A).

## Discussion

In this study, we demonstrated the much higher transduction efficiency of Y444F ssAAV2 to RGCs, especially to Müller cells compared to Wt ssAAV2. With Y444F ssAAV2 mediated shRNA targeting PTEN, RGCs survival and axons regeneration was significantly induced through activating PTEN/mTOR pathway and regulating the expression of GLAST after ONA in rats.

RGCs apoptosis, as the final result of traumatic optic neuropathy, should be prevented and rescued before the irreversible vision loss. More and more evidence has supported the promise of AAV-mediated gene therapy for the treatment of optic neuropathy [10]. However, the ubiquitin-proteasome degradation process has been considered a critical obstacle that inhibits AAV-mediated gene expression by degrading the viral particles during their intracellular trafficking from the cytoplasm to nucleus, which arises from the phosphorylation at tyrosine residues by epidermal growth factor receptor protein tyrosine kinase [25–26]. Y444F scAAV2 has been reported as one of the most efficient single point mutants [16–17], with which the exposed tyrosine (Y) residue 444 on AAV2 capsid is replaced by phenylalanine (F). Y to F mutation has been reported to be able to circumvent phosphorylation and subsequent ubiquitination, leading to higher transduction efficiency both *in vitro* and *in vivo* [16–17, 27]. But the reduced packaging capacity of scAAV2 represents a significant disadvantage for many gene-delivery applications. In this study, we verified the more potent transduction efficiency of Y444F ssAAV2, making it the more suitable deliver tool with large capacity for treating traumatic optic neuropathy.

The PI3K/AKT/mTOR signaling pathway has been implicated in neuron survival and neurite outgrowth [5, 28–29]. mTOR, down-regulated in CNS development and further reduced after axotomy [6, 30], plays a crucial role in modulating protein synthesis, axons growth in development and in response to injury [31–33]. PTEN dephosphorylates phosphatidylinositol 3, 4, 5-trisphosphate into phosphatidylinositol 4, 5-bisphosphate and hence antagonizes the PI3K/AKT signaling. Thus, inactivating PTEN activates Akt and mTOR, facilitates the RGCs survival after optic neuropathy. Our study showed that Y444F AAV2-shRNA.PTEN-GFP led to about 55% reduction in PTEN expression in entire retina while Wt AAV2-shRNA.PTEN-GFP led to about 29% reduction. Correspondingly, Y444F AAV2-shRNA.PTEN-GFP promoted the activity of mTOR complex-1 of retina more significantly than Wt AAV2-shRNA.PTEN-GFP, as pS6 was found up-regulated more significantly with Western blotting and immunofluorescence.

Another interesting finding of our study is that Y444F ssAAV2 could also transduce Müller cells, while Y444F scAAV2 vector is found to be unable to transduce Müller cells via intravitreal injection [27]. Müller cell is the principal glial cell of the vertebrate retina, spans the entire thickness of retina, serves numerous important physiological functions in the retina, including supporting neurons metabolism, maintaining the homeostasis of the retinal extracellular environment, scavenging free radicals, protecting neurons via a release of neurotrophic factors, recycling neurotransmitters, and secreting antioxidants [34–35]. Müller cell can survive under neurodegenerative conditions, be activated by virtually all pathogenic stimuli. Reactive Müller cells can support the survival of photoreceptors and neurons [36], may mediate independent protection of the entire retina for a long period, thus they are thought to be an ideal target for viral gene therapy for neuroprotection [34]. Expressed in Müller cells, GLAST is the prominent glutamate transporter within the retina removing about 50% extracellular glutamate for the prevention of neurotoxicity [37]. Previous and more recent study has verified that optic nerve injury leads to the retinal extracellular glutamate rising to neurotoxic level [38–39]. Our data showed the higher protein expression of GLAST 6 weeks after ONA in Y444F AAV2-shRNA.PTEN-GFP group than in Wt AAV2-shRNA.PTEN-GFP group and Wt AAV2GFP group, which was in line with that Müller cells was transduced. The up-regulation of GLAST is one of anti-apoptotic approaches in the adult CNS and the PI3K pathway is a pivotal component in GLAST up-regulation [40]. We thought PI3K activation via PTEN knockdown in transduced Müller cells stimulated GLAST expression and the higher GLAST expression might act as an extra contributor to RGCs survival. The mechanisms underlying this protective effect are that GLAST activity triggers Ca^2+^ influx, increases mTOR activity, and activates activator protein-1 binding to DNA [41], in addition to avoiding excitotoxicity [42].

RGCs survival is prerequisite for axons regeneration, but axons regeneration is not inevitable in adults in the absence of specific axogenic stimuli [43]. Activation of the mTOR pathway was reportedly sufficient to promote both RGCs survival and axons regeneration [5]. More recent study further indicates that mTOR complex-1 is necessary but mTOR complex-2 and GSK3b are inhibitory for AKT3-induced axons regeneration in CNS [44]. In our study, we observed that Y444F AAV2-shRNA.PTEN-GFP led to more robust axons regeneration and considerable regenerating axons regrew along optic nerve back to optic chiasm while Wt AAV2-shRNA.PTEN-GFP resulted in limited axons regeneration and no regenerating axon could reach optic chiasm. Other recent study also shows that conventional AAV2 mediated-shRNA suppression of PTEN does not lead to axons regeneration back to optic chiasm [45]. This difference could attribute to the different extent of the mTOR complex-1 activation and GLAST regulation resulted from the transduction of RGCs and Müller cells.

In summary, our findings revealed that Y444F ssAAV2 mediated-PTEN knockdown would activate mTOR complex-1 and induce long-distance optic nerve fiber regeneration in wild-type animals, which presented a translatable treatment for traumatic optic neuropathy. In view of complex balancing mechanism implicated in axon regeneration, future studies will be carry out towards combining regulation of mTOR complex-1 and other crucial targets to achieve robust enough axon regeneration for functional vision recovery.

## Supporting information

S1 Dataset The relevant data of the expriments involved in this study. (DOCX)
