# Supplementary material for: Tyrosine-mutated AAV2-mediated shRNA silencing of PTEN promotes axon regeneration of adult optic nerve
Source: PLoS One. 2017 Mar 21;12(3):e0174096. doi: 10.1371/journal.pone.0174096 (PMC5360277; doi:10.1371/journal.pone.0174096)
Supplement: S1 Dataset — (DOCX) [file pone.0174096.s001.docx]

**Dataset 1. The ratio of the mean density of GFP(+) cells related to that of Fluo(+) cells in wt AAV2 or Y444F AAV2 group.**

| **WT-AAV2 group** | | | | **Y444F-AAV2 group** | | |
| --- | --- | --- | --- | --- | --- | --- |
| n | GFP+( /mm^2^) | Fluo+( /mm^2^) | ratio | GFP+( /mm^2^) | Fluo+( /mm^2^) | ratio |
| 1 | 1705 | 3564 | 0.478437 | 3738 | 3764 | 0.99306 |
| 2 | 2440 | 3128 | 0.77997 | 3136 | 3910 | 0.801978 |
| 3 | 1465 | 4012 | 0.3652 | 2820 | 3410 | 0.826835 |
| 4 | 2004 | 3902 | 0.513565 | 3441 | 3879 | 0.887164 |
| 5 | 1225 | 3682 | 0.332701 | 3478 | 4010 | 0.867255 |
| 6 | 1813 | 3762 | 0.481983 | 3174 | 3530 | 0.899261 |

**Dataset 2. The individual value of fluorescence intensity of PTEN in GCL of retinas 4 weeks after intravitreal injection of AAV2 vectors.**

| n | **Wt AAV2-GFP group** | **Wt AAV2-shRNA.PTEN-GFP group** | **Y444F AAV2-shRNA.PTEN-GFP group** |
| --- | --- | --- | --- |
| 1 | 249.369 | 83.128 | 52.135 |
|  | 231.437 | 78.044 | 42.698 |
|  | 190.093 | 49.861 | 40.249 |
|  | 126.821 | 165.121 | 32.654 |
|  | 124.891 | 113.141 | 22.237 |
|  | 202.913 | 79.027 | 28.981 |
| 2 | 86.578 | 122.029 | 43.482 |
|  | 233.038 | 55.051 | 47.293 |
|  | 101.831 | 111.397 | 55.258 |
|  | 120.983 | 96.037 | 59.159 |
|  | 104.191 | 167.931 | 24.357 |
|  | 210.526 | 62.235 | 41.767 |
| 3 | 212.018 | 76.121 | 63.128 |
|  | 272.534 | 68.097 | 56.169 |
|  | 96.218 | 42.124 | 44.463 |
|  | 171.025 | 148.337 | 51.753 |
|  | 105.91 | 103.008 | 30.951 |
|  | 219.663 | 89.091 | 19.851 |
| 4 | 178.568 | 135.397 | 29.973 |
|  | 157.431 | 54.567 | 45.853 |
|  | 99.028 | 130.131 | 44.716 |
|  | 79.769 | 95.021 | 65.946 |
|  | 113.287 | 161.054 | 55.967 |
|  | 169.879 | 62.061 | 22.805 |
| 5 | 165.221 | 84.005 | 40.537 |
|  | 159.212 | 67.531 | 61.939 |
|  | 73.851 | 69.893 | 33.028 |
|  | 88.632 | 148.005 | 24.361 |
|  | 99.457 | 104.187 | 29.029 |
|  | 203.125 | 88.369 | 51.568 |

**Dataset 3. The individual value of fluorescence intensity of PTEN in INL of retinas 4 weeks after intravitreal injection of AAV2 vectors.**

| n | **Wt AAV2-GFP group** | **Y444F AAV2-shRNA.PTEN-GFP group** | **Wt AAV2-shRNA.PTEN-GFP group** |
| --- | --- | --- | --- |
| 1 | 22.036 | 7.036 | 13.036 |
|  | 21.076 | 9.391 | 14.055 |
|  | 18.556 | 12.125 | 10.705 |
|  | 14.769 | 6.913 | 17.571 |
|  | 15.171 | 10.378 | 14.099 |
|  | 12.451 | 8.171 | 15.546 |
| 2 | 25.194 | 6.159 | 21.057 |
|  | 21.508 | 5.502 | 13.252 |
|  | 20.139 | 8.789 | 15.258 |
|  | 17.756 | 5.852 | 15.697 |
|  | 24.097 | 7.369 | 13.087 |
|  | 21.631 | 8.661 | 16.954 |
| 3 | 16.432 | 9.099 | 10.423 |
|  | 18.739 | 6.025 | 17.087 |
|  | 14.697 | 9.075 | 18.251 |
|  | 13.252 | 9.259 | 14.369 |
|  | 27.312 | 6.415 | 21.771 |
|  | 19.539 | 5.625 | 11.208 |
| 4 | 17.369 | 8.098 | 12.331 |
|  | 18.715 | 6.021 | 16.089 |
|  | 20.951 | 7.036 | 12.924 |
|  | 21.067 | 8.036 | 11.715 |
|  | 19.526 | 11.664 | 10.409 |
|  | 15.923 | 6.381 | 19.936 |
| 5 | 19.397 | 10.367 | 12.973 |
|  | 12.158 | 8.028 | 16.068 |
|  | 28.025 | 7.366 | 19.903 |
|  | 20.507 | 5.309 | 14.253 |
|  | 18.004 | 6.313 | 15.285 |
|  | 17.754 | 6.039 | 18.429 |

**Dataset 4. The ratio of the expression of PTEN related to that of GAPDH in retina 4 weeks after intravitreal injection of AAV2 vectors examined with Western-blotting.**

| n | Intact Control | Wt AAV2-GFP | Y444F AAV2-shRNA.PTEN-GFP | Wt AAV2-shRNA.PTEN-GFP |
| --- | --- | --- | --- | --- |
|  | PTEN/GAPDH | PTEN/GAPDH | PTEN/GAPDH | PTEN/GAPDH |
| 1 | 0.603171 | 0.573094 | 0.299284 | 0.479168 |
| 2 | 0.692243 | 0.696105 | 0.302765 | 0.506939 |
| 3 | 0.634306 | 0.765739 | 0.27081 | 0.386646 |
| 4 | 0.659906 | 0.684997 | 0.310952 | 0.427584 |
| 5 | 0.609905 | 0.604979 | 0.270953 | 0.437579 |

**Dataset 5. The ratio of the expression of ps6 related to that of GAPDH in retina 4 weeks after intravitreal injection of AAV2 vectors examined with Western-blotting.**

| **n** | **Intact Control** | **Wt AAV2-GFP** | **Y444F AAV2-shRNA.PTEN-GFP** | **Wt AAV2-shRNA.PTEN-GFP** |
| --- | --- | --- | --- | --- |
|  | ps6/GAPDH | ps6/GAPDH | ps6/GAPDH | ps6/GAPDH |
| 1 | 0.391348 | 0.352093 | 0.602434 | 0.490002 |
| 2 | 0.320769 | 0.439532 | 0.802012 | 0.573346 |
| 3 | 0.496385 | 0.33239 | 0.60049 | 0.453398 |
| 4 | 0.20947 | 0.23146 | 0.90172 | 0.41139 |
| 5 | 0.393243 | 0.300119 | 0.651664 | 0.482034 |

**Dataset 6. The ratio of the expression of GLAST related to that of GAPDH in retina 4 weeks after intravitreal injection of AAV2 vectors examined with Western-blotting.**

|  | **Intact control** | **Wt AAV2-GFP** | **Y444F AAV2-shRNA.PTEN-GFP** | **Wt AAV2-shRNA.PTEN-GFP** |
| --- | --- | --- | --- | --- |
| n | GLAST/GAPDH | GLAST/GAPDH | GLAST/GAPDH | GLAST/GAPDH |
| 1 | 0.676627 | 0.665146 | 0.811927 | 0.861341 |
| 2 | 0.628871 | 0.673478 | 0.85873 | 0.723419 |
| 3 | 0.78713 | 0.739693 | 0.601918 | 0.668879 |
| 4 | 0.557826 | 0.74443 | 0.816529 | 0.735134 |
| 5 | 0.662613 | 0.705687 | 0.747276 | 0.734944 |

**Dataset 7. The ratio of the expression of GLAST related to that of GAPDH in retina 6 weeks after axotomy of each group examined with Western-blotting.**

| **n** | **Intact Control** | **Wt rAAV2-GFP** | **Y444F rAAV2-shRNA.PTEN-GFP** | **Wt rAAV2-shRNA.PTEN-GFP** |
| --- | --- | --- | --- | --- |
|  | GLAST/GAPDH | GLAST/GAPDH | GLAST/GAPDH | GLAST/GAPDH |
| 1 | 1.13688 | 0.128158 | 0.330765 | 0.10582 |
| 2 | 0.981079 | 0.097275 | 0.23723 | 0.129024 |
| 3 | 0.894513 | 0.126249 | 0.307767 | 0.099167 |
| 4 | 0.974169 | 0.101894 | 0.281931 | 0.101337 |
| 5 | 0.884158 | 0.110901 | 0.251922 | 0.141336 |

**Dataset 8. The number of PS6(+) cells in 100 RGCs per retina section 4 weeks after intravitreal injection of AAV2 vectors.**

| n | Wt AAV2-GFP | Wt AAV2-shRNA.PTEN-GFP | Y444F AAV2-shRNA.PTEN-GFP |
| --- | --- | --- | --- |
| 1 | 8 | 12 | 21 |
|  | 6 | 15 | 24 |
|  | 9 | 16 | 23 |
|  | 8 | 10 | 33 |
|  | 10 | 29 | 18 |
|  | 5 | 20 | 23 |
| 2 | 11 | 19 | 18 |
|  | 5 | 15 | 28 |
|  | 2 | 18 | 19 |
|  | 8 | 17 | 25 |
|  | 5 | 16 | 28 |
|  | 7 | 20 | 21 |
| 3 | 8 | 12 | 21 |
|  | 7 | 18 | 22 |
|  | 9 | 14 | 24 |
|  | 7 | 11 | 34 |
|  | 12 | 27 | 19 |
|  | 5 | 20 | 23 |
| 4 | 10 | 18 | 17 |
|  | 5 | 16 | 29 |
|  | 2 | 20 | 20 |
|  | 8 | 17 | 25 |
|  | 5 | 18 | 28 |
|  | 6 | 16 | 19 |
| 5 | 8 | 12 | 20 |
|  | 7 | 18 | 22 |
|  | 10 | 15 | 24 |
|  | 7 | 10 | 30 |
|  | 11 | 26 | 19 |
|  | 5 | 21 | 23 |
| 6 | 12 | 19 | 19 |
|  | 4 | 15 | 26 |
|  | 3 | 16 | 20 |
|  | 8 | 17 | 25 |
|  | 5 | 18 | 28 |
|  | 7 | 20 | 19 |

**Dataset 9. The density of TUJ1 positive RGCs of each group at 6 weeks after axotomy (/mm^2^).**

|  | **Wt AAV2-shRNA.PTEN-GFP** | | **Y444F AAV2-shRNA.PTEN-GFP** | | **Wt AAV2-GFP** | |
| --- | --- | --- | --- | --- | --- | --- |
| n | Intact control | ONA6w | Intact control | ONA6w | Intact control | ONA6w |
| 1 | 2613 | 508 | 2700 | 754 | 2788 | 247 |
| 2 | 3020 | 853 | 3222 | 1122 | 2499 | 122 |
| 3 | 2514 | 472 | 2222 | 930 | 3120 | 223 |
| 4 | 2227 | 420 | 2781 | 953 | 2524 | 141 |
| 5 | 2472 | 553 | 2255 | 802 | 2469 | 271 |

**Dataset 10. The fluorescence intensity values at different distances proximal to and distal to the crush site of optic nerve of each group.**

|  | **Y444F AAV2-shRNA.PTEN-GFP** | | | | | **Wt AAV2-shRNA.PTEN-GFP** | | | | | | **Wt AAV2-GFP** | | | | |
| --- | --- | --- | --- | --- | --- | --- | --- | --- | --- | --- | --- | --- | --- | --- | --- | --- |
| n | proximal | distal 0.5mm | distal 1.5mm | distal 2.5mm | distal 3.5mm | proximal | distal 0.5mm | distal 1.5mm | distal 2.5mm | distal 3.5mm | proximal | | distal 0.5mm | distal 1.5mm | distal 2.5mm | distal 3.5mm |
| 1 | 39.855 | 10.324 | 9.397 | 10.18 | 9.07 | 14.286 | 7.967 | 5.469 | 7.675 | 4.696 | 3.171 | | 2.3 | 1.772 | 2.422 | 2.667 |
| 2 | 37.608 | 9.392 | 8.464 | 10.857 | 9.092 | 29.656 | 4.429 | 6.036 | 5.663 | 5.915 | 2.372 | | 1.746 | 1.833 | 1.861 | 1.638 |
| 3 | 34.986 | 7.128 | 6.869 | 7.613 | 6.118 | 25.944 | 5.209 | 4.447 | 4.908 | 6.263 | 2.442 | | 2.69 | 1.485 | 1.564 | 1.516 |
| 4 | 32.398 | 8.912 | 9.128 | 9.782 | 7.234 | 29.011 | 6.342 | 3.288 | 3.563 | 3.3874 | 2.833 | | 2.093 | 1.783 | 1.782 | 1.832 |
| 5 | 24.781 | 13.435 | 6.876 | 6.879 | 5.986 | 18.382 | 4.231 | 2.902 | 2.984 | 3.0981 | 1.988 | | 1.988 | 1.783 | 2.061 | 1.984 |
